# *Magel2*, a Prader-Willi syndrome candidate gene, modulates the activities of circadian rhythm proteins in cultured cells

**DOI:** 10.1186/1740-3391-9-12

**Published:** 2011-12-30

**Authors:** Julia Devos, Sara V Weselake, Rachel Wevrick

**Affiliations:** 1 Department of Medical Genetics, University of Alberta, Edmonton, AB Canada T6G 2H7

**Keywords:** suprachiasmatic nucleus, Prader-Willi syndrome, luciferase assay, melanoma antigen gene, necdin

## Abstract

**Background:**

The Magel2 gene is most highly expressed in the suprachiasmatic nucleus of the hypothalamus, where its expression cycles in a circadian pattern comparable to that of clock-controlled genes. Mice lacking the *Magel2 *gene have hypothalamic dysfunction, including circadian defects that include reduced and fragmented total activity, excessive activity during the subjective day, but they have a normal circadian period. Magel2 is a member of the MAGE family of proteins that have various roles in cellular function, but the specific function of Magel2 is unknown.

**Methods:**

We used a variety of cell-based assays to determine whether Magel2 modifies the properties of core circadian rhythm proteins.

**Results:**

Magel2 represses the activity of the Clock:Bmal1 heterodimer in a Per2-luciferase assay. Magel2 interacts with Bmal1 and with Per2 as measured by co-immunoprecipitation in co-transfected cells, and exhibits a subcellular distribution consistent with these interactions when visualized by immunofluorescence. As well, Magel2 induces the redistribution of the subcellular localization of Clock towards the cytoplasm, in contrast to the nucleus-directed effect of Bmal1 on Clock subcellular localization.

**Conclusion:**

Consistent with the blunted circadian rhythm observed in *Magel2-*null mice, these data suggest that Magel2 normally promotes negative feedback regulation of the cellular circadian cycle, through interactions with key core circadian rhythm proteins.

## Introduction

Prader-Willi syndrome (PWS) is a genetic neurodevelopmental disorder featuring neonatal failure to thrive, hyperphagia leading to obesity, growth hormone deficiency, and other findings [1-3]. About 80% of affected individuals suffer from sleep-wake cycle disturbances, typically characterized as excessive daytime sleepiness, with night or early morning waking [4]. These clinical findings implicate hypothalamic dysfunction in PWS, but the pathophysiology of this disorder remains poorly understood, in part because at least five genes are inactivated in typical affected individuals. We identified *MAGEL2*, a member of the necdin/MAGE protein family, as a candidate gene for some features of PWS [2,3]. In adult mice, *Magel2 *expression is restricted to the nervous system, with the majority of *Magel2*-positive neurons located in the hypothalamus [5]. Within the hypothalamus, *Magel2 *is primarily expressed in the paraventricular nucleus, supraoptic nucleus, and in the suprachiasmatic nucleus (SCN), which is the circadian rhythm generating center of the brain [6]. Within the SCN, *Magel2*-positive neurons overlap with neurons expressing arginine vasopressin in the dorsal SCN, which modulate circadian rhythm and conduct circadian output signals to target organs and other areas of the brain.

Using running wheel activity monitoring, we previously found that gene-targeted *Magel2*-deficient mice had reduced and more fragmented total activity, with a diminished circadian amplitude [7]. They also had more activity during the subjective day compared to wild-type mice, but had a normal circadian period. We also discovered progressive infertility in these mice, with irregular estrus cycles in female mice that mimic those seen in other circadian mutant mice [8,9]. These and other findings of endocrine disruption [10] support a role for loss of MAGEL2 in the hypothalamic disruption observed in PWS. However, the manner in which Magel2 normally contributes to the maintenance of circadian rhythm in hypothalamic neurons has not been explored.

The expression of many genes in the SCN follows a circadian cycle, such that different groups of genes have peaks of high expression at specific times of the 24-hour day. Within each cell, a roughly 24-hour cycle of gene expression is driven by a transcription translation feedback loop that results in the periodic transcription of a set of genes, the clock-controlled genes. This loop is driven by the activity of a heterodimer of two bHLH (basic helix-loop-helix) transcription factors, Clock and Bmal1. Clock and Bmal1 heterodimerize to activate clock-controlled genes, typically acting through E-box elements in the target promoters [11]. These target genes include those of the Period (Per) and Cryptochrome (Cry) families. The proteins encoded by the *Per *and *Cry *genes accumulate, then heterodimerize and feedback to inhibit the activity of Clock:Bmal1 and thus their own transcription, which reaches a nadir by the end of circadian night [12,13]. A delay in this negative feedback loop is introduced by the regulated post-translational modification and E3-ligase-mediated degradation of Cry and Per in the cytoplasm [14,15].

In a thorough examination of gene expression in the SCN of mice entrained to a 12 hour light-12 hour cycle dark, then released into darkness, 337 genes were found to have circadian expression in the SCN [6]. Among these, *Magel2 *was one of the most highly circadian genes identified, with *Per2 *also identified as expected. The peak of *Magel2 *expression is at circadian time (CT) 12-14, in the late light period, and the trough at CT24, a pattern that was verified by *in situ *hybridization in additional mouse SCN samples [6,7]. This expression peak is about 2 hours delayed from that of *Per2*, which has peak expression around CT10, whereas the peak of Clock:Bmal1 activity is around CT8 [16]. The circadian pattern of expression and the timing of peak expression suggest that, like *Per2*, *Magel2 *is a clock-controlled gene, although this has not been directly demonstrated.

The physiological mechanism of action of Magel2 in the maintenance of circadian rhythm is unknown. Magel2 is one of the lesser studied of a family of "MAGE" proteins, which contain a melanoma antigen (MAGE) homology domain that contributes to protein-protein interactions [17,18]. MAGE proteins can interact with transcription factors and nuclear receptors, with downstream effects on gene activity. Notably, previous studies have demonstrated physical interactions between the MAGE protein necdin and proteins of the bHLH-PAS (Per/Arnt/Sim) family of transcription factors [19,20]. These necdin-interacting proteins include Hif1α, Arnt (Hif1β), Arnt2, and Bmal1, with the interactions occurring through the bHLH domain of each of the bHLH-PAS transcription factors. The heterodimers Arnt:Hif1α and Arnt2:Sim1 activate transcription by binding upstream response elements in target genes, and co-expression of necdin represses this activity [19,20]. Necdin also interacts with SIRT1, which regulates the amplitude of clock-controlled gene expression through deacetylation of Per2, Bmal1, and associated histones [21-23].

MAGE proteins also modulate the activity of E3 ubiquitin ligases through their interactions with RING domain proteins, forming complexes that regulate protein degradation. A multitude of such interactions have been uncovered through interaction screens and directed assays, and the large number of MAGE proteins (~60) and of RING domain proteins (hundreds) makes additional undiscovered interactions likely [18,24-28]. MAGE proteins modulate protein stability by recruiting E2 conjugating enzymes to specific E3 substrate complexes. Candidate target proteins for MAGE/RING mediated degradation include p53 (MAGE/TRIM28) [26], AATF/Che1 (MAGED1/RING protein) [29] Hif1α (necdin/RING protein, possibly Rbx1/Roc1) [19,30], EID1 (necdin/RING protein) [31], and Fez1 (necdin or Magel2/RING protein), [32]. Many of the proteins targeted by E3 ligases are regulated by sequestration and proteosomal degradation in the cytoplasm, in some cases through their interactions with MAGE proteins [26,27,29,31,33]. With respect to core circadian proteins, the Skp1-Cullin-F box E3 ligase-associated proteins are implicated in the targeted degradation of Cry and Per proteins [14,15], but the RING protein associated with the complex, namely Rbx1/Roc1 [30], has not been yet been associated with a specific MAGE protein.

Lastly, mice deficient for the MAGE gene *Maged1 *exhibit a short period circadian phenotype, but *Maged1 *itself is not circadian in its expression [34]. It was proposed that Maged1 normally cooperates with the orphan nuclear receptor Rorα to modulate the expression of circadian genes, including *Bmal1*. Altogether, these results suggest multiple ways in which MAGE proteins could act in a feedback loops that regulate the rhythmic activity of circadian proteins.

The circadian profile of *Magel2*, the participation of MAGE proteins in the regulation of unstable proteins and transcription factors, and the circadian phenotype in the *Magel2*-null mice, together strongly suggest that *Magel2 *is involved in regulation of circadian rhythm. We therefore hypothesized that Magel2 could interact with core circadian rhythm proteins and modify their activity, acting to regulate either the transcriptional function or the subcellular localization of these proteins. We examined the effect of Magel2 on circadian rhythm proteins, in a heterologous expression system in tissue culture cells. We found that indeed Magel2 represses Clock:Bmal1 mediated transcription, and that Magel2 can interact with Per2 and with Bmal1. Interestingly, Magel2 co-localizes with Clock and Bmal1 in the cytoplasm, and with Per2 in the cytoplasm and nucleus, and Magel2 influences the subcellular localization of Bmal1 and Clock. These results lend support to the importance of Magel2 in the regulation of circadian proteins, as suggested from the circadian phenotype of *Magel2-*null mice.

## Methods

### Plasmids

The previously described epitope-tagged expression plasmid for murine Magel2 (pcDNA4HisMaxXpressMagel2) is referred to as pXpress-Magel2 [32]. Expression constructs for murine Per2 (pcDNA3.1Per2V5His, pPer2-V5) and Cry1 (pcDNA3.1Cry1V5His, pV5-Cry) were obtained from Dr. J. Takahashi at Northwestern University [35]. An expression plasmid for murine Bmal1 (pcDNA3HABmal1, pHA-Bmal1) and two murine Clock constructs (pcDNA3HAClock, pHA-Clock and pcDNA3.1MycClock, pMyc-Clock) were obtained from Dr. M. Antoch at the Roswell Park Cancer Institute [36,37]. The pGL3-E2 box circadian enhancer plasmid, mPer2:luc, includes the noncanonical circadian enhancer E-box from *Per2 *and was from Dr. J. Takahashi [38]. A pRL-TK *Renilla *luciferase vector was used as an internal control reporter [31].

### Antibodies

Primary antibodies used for immunoblotting and immunofluorescence were: mouse monoclonal anti-Xpress and anti-V5 (Invitrogen, Carlsbad, CA), mouse monoclonal anti-HA (Sigma-Aldrich, Oakville, ON), rabbit polyclonal anti-HA (Santa Cruz Biotechnologies, Santa Cruz, CA), rabbit polyclonal anti-Myc (Sigma-Aldrich), mouse monoclonal anti-myc (Sigma), and rabbit polyclonal anti-V5 (Millipore, Billerica, MA). Secondary antibodies used for immunoblots were sheep anti-mouse and goat anti-rabbit IgG (GE Healthcare). Secondary antibodies used for immunofluorescence were goat anti-mouse Alexa Fluor 488 and 594 and goat anti-rabbit Alexa Fluor 488 and 594 (Invitrogen).

### Cell culture and transient transfection

NIH3T3 cell lines were cultured in Dulbecco's modified Eagle's medium (DMEM) supplemented with 10% calf serum (CS) (Invitrogen). HEK293 cell lines were maintained in DMEM supplemented with 10% fetal bovine serum. NIH3T3 or HEK293 cells were plated 18 to 24 hours prior to transfection, then transfected using Attractene Transfection Reagent (Qiagen, Mississauga, ON) according to the manufacturer's instructions. For the luciferase assays, a total of 0.4 μg of appropriate combination of plasmid DNA and 5 ng of pRL-TK *Renilla *luciferase control plasmid were diluted in 60 μL of DMEM. One μL of Attractene was added to the diluted DNA and mixed by light tapping. When required, vector plasmids without cDNA inserts were co-transfected in order to keep the total amount of plasmid constant across all transfections. The resulting mixtures were incubated for 15 min. at room temperature. The appropriate plasmid combinations were added drop-wise onto the cells and the plates swirled to evenly distribute the mixture. The plates were then incubated at 37°C and 5% CO_2 _for 18 to 24 hours before recovering cell lysates for immunoblotting or luciferase assays. Alternatively, cells were plated into 6 well dishes with coverslips, transfected, then the coverslips were processed for immunohistochemistry. Protein half-life was measured by treating cells with 100 μg/ml cycloheximide (Sigma-Aldrich). Cell lysates were then collected and immunoblotting was used to detect and quantify protein levels, which were then plotted versus the collection times. Co-immunoprecipitations, SDS-polyacrylamide gel electrophoresis and immunoblotting were used to separate and visualize proteins, essentially as described [31]. Decay curves were fit to the data points, and the half-life of each recombinant protein determined using the equation of the line. Immunoblot analysis was performed using Kodak Image Station Software and protein quantities were calculated as a ratio of the pixel intensity from the protein of interest to that of γ-tubulin.

### Dual luciferase assays

Dual luciferase assays were performed using the Dual Luciferase Reporter System kit (Promega Corporation, Madison, WI). Relative luciferase activity was assayed as previously described using a TD-20/20 Luminometer (Turner Designs, Sunnyvale, CA) and normalized to an internal *Renilla *luciferase control [31].

### Immunofluorescence

Cells were plated on coverslips in a 6-well plate and transfected. The next day, the cells were washed with PBS, fixed in 4% paraformaldehyde, then washed three times in PBS with 0.05% Triton-X (PBSX). Nonspecific antibody binding was blocked with 2% Blocking Reagent (Roche Applied Science, Laval, QC) for 15 min. The coverslips were then incubated with the appropriate primary antibodies for 1 hour at room temperature, washed twice with PBSX, incubated for 1 hour with the appropriate fluorescently tagged secondary antibody, washed twice in PBSX. Hoechst 33342 was added to stain nucleic acids, the coverslips were washed again in PBSX, then were mounted on glass slides. Triple fluorescence images were acquired then processed in ImageJ64 (imagej.nih.gov/ij) and analyzed using the PlotProfile function for nuclear-cytoplasmic subcellular localization and signal overlap. Linescan diagrams from individual representative co-transfected cells are shown, with the Hoechst fluorescence in blue.

### Statistical analysis

**Figure 1:**
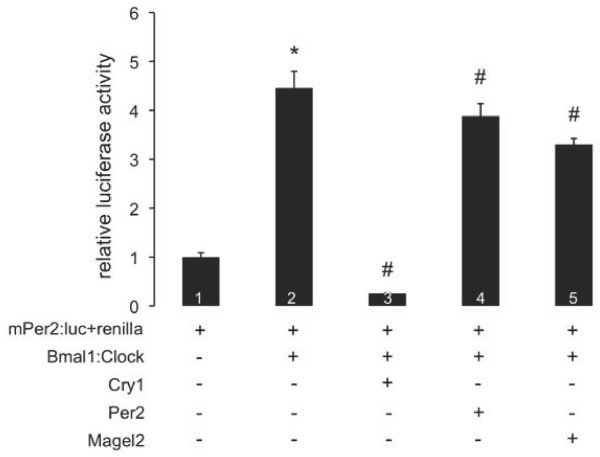
**Magel2 represses Clock:Bmal1-mediated transcription at an E-box promoter**. Dual luciferase transcription assays following transfection of NIH3T3 cells with the mPer2:luc reporter plasmid and a renilla internal control plasmid, together with combinations of expression plasmids variously encoding epitope-tagged Clock, Bmal1, Magel2, Cry1, and Per2 proteins. Data are normalized to the baseline activity of the normalized mPer2:luc reporter. A * indicates a significant difference from the baseline mPer2:luc activity, and a # indicates a significant repression compared to Bmal1:Clock-mediated activation (*p *< 0.05). These results represent one of three experiments completed in triplicate. Error bars represent the standard error of the mean.

Luciferase assays were performed in triplicate, with one representative set shown in Figure 1. Values indicating the mean +/- the standard error of the mean and compared between assays for significance at *p *< 0.05 using the Student t-test. Values obtained from counts of cells with predominantly nuclear or predominantly cytoplasmic localization of various proteins were analyzed for differences using a Fisher exact test.

## Results

### Expression of Magel2 reduces Clock:Bmal1 heterodimer activity at the *Per2 *promoter

Modulation of circadian rhythm by Magel2 could involve regulation of Clock:Bmal1-mediated transcription, which can be measured by ectopic expression of reporter constructs in tissue culture cells [38]. The reporter plasmid (*mPer2-E2:Luciferase*, mPer2:luc) containing a circadian E-box enhancer (E2) was activated 4.5-fold by Clock:Bmal1, as expected (Figure 1, assay 2 versus assay 1). Co-expression of either Cry1 or Per2 repressed Clock:Bmal1-mediated activation of mPer2:luc (assays 3 and 4 versus assay 2). Co-expression of Cry1, a stronger repressor than Per2, decreased relative luciferase activity significantly below baseline (assay 1) levels, likely because Cry1 is also inhibiting the activity of endogenous Clock:Bmal1 on the mPer2:luc construct. In turn, Magel2 also significantly repressed activation by Clock:Bmal1 (assay 5 versus assay 2). Repression by Magel2 was intermediate in strength between that of the more repressive Cry1 and the less repressive Per2. These results suggest that Magel2 can act as a negative regulator of Clock/Bmal1-mediated transcription.

### Magel2 interacts with Bmal1 and Per2

**Figure 2:**
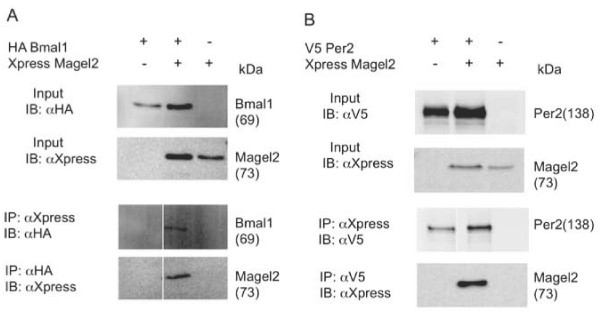
**Magel2 interacts with Bmal1 and Per2**. A) HEK293 cells were transfected with expression plasmids encoding HA-Bmal1, Xpress-Magel2, or both constructs. Five percent of the volume of cell lysate used for immunoprecipitation was immunoblotted to confirm the presence of HA-Bmal1 and Xpress-Magel2 protein in the appropriate input samples (top). The protein complexes were immunoprecipitated with anti-HA or anti-Xpress antibodies, and the immunoprecipitates detected by immunoblotting with antibodies directed against the reciprocal epitope tag (bottom). B) HEK293 cells were transfected with expression plasmids encoding V5-Per2, Xpress-Magel2, or both constructs. Five percent of the volume of cell lysate used for immunoprecipitation was immunoblotted to confirm the presence of Xpress-Magel2 and V5-Per2 protein in the appropriate input samples (top). The protein complexes were immunoprecipitated with anti-V5 or anti-Xpress antibodies, and the immunoprecipitates detected by immunoblotting with antibodies directed against the reciprocal epitope tag (bottom).

We next tested whether Magel2 interacts with Bmal1 or Per2 by co-immunoprecipitating epitope-tagged proteins in transfected HEK293 cells (Figure 2). Protein lysates from cells co-transfected with pXpress-Magel2 and pHA-Bmal1 were immunoblotted to confirm the presence of both pXpress-Magel2 and pHA-Bmal1 in the appropriate samples (Figure 2A). We immunoprecipitated the same lysate with anti-HA antibodies (to immunoprecipitate Bmal1), then immunoblotted with anti-Xpress to detect Magel2. We only detected immunoprecipitated Xpress-Magel2 in the sample with co-expressed HA-Bmal1, and not in the sample transfected with empty vector control. In the reciprocal experiment, immunoprecipitation of Xpress-Magel2 also co-immunoprecipitated HA-Bmal1. Likewise, we co-transfected pXpress-Magel2 and pV5-Per2 into HEK293 cells, and detected interactions by co-immunoprecipitation (Figure 2B). In this case, in an immununoprecipitation with anti-V5, Xpress Magel2 was only detected in the presence of co-expressed V5-Per2. While we did detect V5-Per2 when we immunoprecipitated Xpress-Magel2, there was also some immunoprecipitation of V5-Per2 in the absence of Xpress-Magel2, which we interpret as nonspecific binding of V5-Per2 to the protein G beads used in the assay.

**Figure 3:**
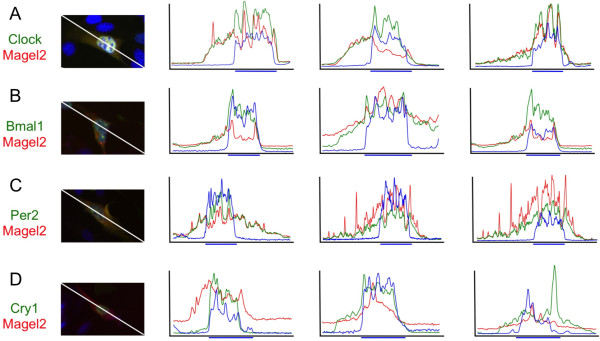
**Subcellular localization of circadian proteins in the presence of Magel2**. pXpressMagel2 and plasmids encoding other epitope-tagged circadian proteins (A, Clock, B, Bmal1, C, Per2, and D, Cry1) were detected in co-transfected NIH3T3 cells by immunofluorescence. Plots of fluorescence intensity were generated to assess overlap between the immunofluorescence signals from each protein within transfected cells, which were also stained with Hoechst 33342 to label the nucleus.

The co-immunoprecipitation experiments show that Magel2 can physically interact with Bmal1, and likely also with Per2. We next examined whether Magel2 co-localized with either protein in transfected cells, by immunofluorescence (Figure 3). We acquired fluorescence images of co-transfected individual cells, using Hoechst staining to visualize the nucleus. We then converted the images to RGB stacks and plotted the fluorescence intensity in lines drawn across individual cells. Fluorescence intensities were acquired in three channels, plotted, and visually examined for overlap in the profiles. The profile of Magel2 fluorescence most resembled that of Clock, with overlapping signals in the cytoplasm in most cells, but also in the nucleus in some cells (e.g. right hand profile in Figure 3A, Magel2/Clock). In about half the cells in which Magel2 and Bmal1 were co-expressed, the fluorescence overlapped in the cytoplasm but overlap was not seen in the nucleus (Figure 3B). We also detected overlapping profiles for Magel2 and Per2, in both the nucleus and the cytoplasm, in some but not all cells (Figure 3C). Lastly, there was no evidence of overlapping profiles in cells co-transfected with Magel2 and Cry1 (Figure 3D).

### Subcellular localization of Bmal1 is altered on co-expression of Magel2

**Figure 4:**
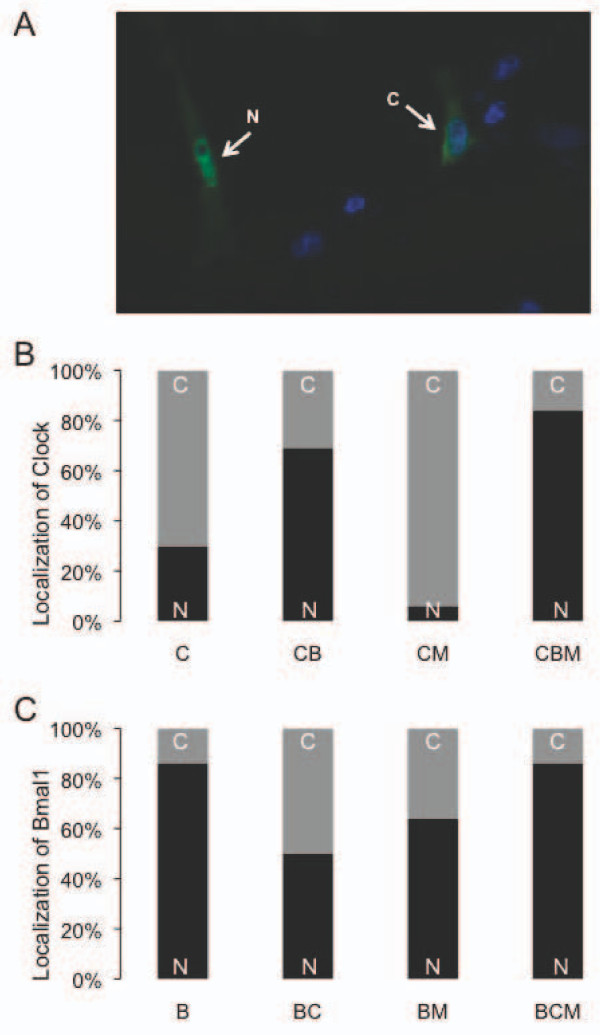
**Subcellular distribution of circadian proteins is modified by co-expression of Magel2**. NIH3T3 cells were transfected with combinations of Xpress-Magel2 and circadian expression constructs. Transfected cells were scored as having the majority of the fluorescent signal either in the nucleus or in the cytoplasm. Differences in the nuclear/cytoplasmic distribution between transfections were detected using a Fisher exact test, with p < 0.05 deemed statistically significant. A) Example of primarily nuclear (N) or primarily cytoplasmic (C) localization of epitope tagged Bmal1 (in green, nucleus is in blue). B) Localization of Clock when expressed alone (C), expressed with Bmal1 (CB), expressed with Magel2 (CM) or expressed with Clock and Magel2 (CBM). C) Localization of Bmal1 when expressed alone (B), expressed with Clock (BC), expressed with Magel2 (BM) or expressed with Clock and Magel2 (BCM).

Bmal1 is regulated by post-translational modifications that alter its stability and subcellular localization. A previous study showed that Bmal1 is predominantly located within the nucleus whether transfected alone or with Clock [36]. We transfected NIH3T3 cells plated on coverslips with various combinations of constructs encoding either epitope-tagged circadian proteins or the empty vector control plasmid, then detected the proteins by immunofluorescence. The subcellular localization of each protein was considered either nuclear or cytoplasmic depending upon the subcellular region where the greatest level of fluorescence was observed, in 100 cells for each plasmid combination (Figure 4A). Consistent with previous results, myc-Clock was predominantly cytoplasmic in most cells when expressed alone (Figure 4B, see also Figure 3A), whereas HA-Bmal1 was predominantly nuclear (Figure 4C, see also Figure 3B). Consistent with the idea that the nuclear accumulation of Clock is Bmal1-dependent, co-expression of Bmal1 in Clock-transfected cells promoted the nuclear accumulation of Clock (Figure 4B). In contrast, Magel2 significantly promoted the cytoplasmic accumulation of Clock. However, when Clock, Bmal1, and Magel2 were all co-expressed, Clock was mostly nuclear, suggesting that Bmal1-induced nuclear shuttling of Clock is more robust than the effect of Magel2. The effect of Magel2 on the subcellular distribution of Bmal1 was less striking: Magel2 induced a redistribution of Bmal1 towards the cytoplasm, an effect that was reversed by co-expression of Clock (Figure 4C). These data suggests that Magel2 can influence the subcellular localization of circadian rhythm proteins. As these proteins are known to vary in their subcellular localization depending on circadian time [36], Magel2 may have different activities depending on the relative abundance of these proteins at different circadian times. In contrast, we found no evidence that Magel2 influences the subcellular localization of Per2 or Cry1 in co-transfected cells.

### Stability of proteins in the presence of Magel2

MAGE proteins interact with RING-domain proteins associated with E3 ligase complexes to modulate proteasomal degradation of specific target proteins. We therefore tested whether expression of varying amounts of Magel2 had any effect on the abundance of co-expressed proteins with which it can interact, including Bmal1 and Per2, in the presence or absence of their heterodimerization partners Clock (for Bmal1) or Cry1 (for Per2). We co-transfected cells with increasing amount of pXpress-Magel2 and a constant amount of pHA-Bmal1, keeping the total amount of plasmid constant with the appropriate empty expression construct backbone. We then immunoblotted separately with anti-HA antibodies to detect Bmal1, and with anti-Xpress antibodies to detect Magel2. We repeated this experiment adding a constant amount of pmyc-Clock, to determine whether any change in stability required the presence of this heterodimerization partner. Similarly, we co-transfected pXpress-Magel2 and pV5-Per2, with or without pV5-Cry1, and quantified these proteins on immunoblots. We also performed experiments using the protein synthesis inhibitor cycloheximide to measure the half-life of these various circadian proteins in the presence or absence of Magel2. We found no evidence for any influence of Magel2 on the stability or abundance of circadian proteins in these co-transfection experiments (data not shown).

## Discussion

The mammalian circadian clock oscillates in a robust, self-sufficient manner. At least two transcription/translation/post-translation feedback loops defend the circadian cycle against random perturbations, while allowing outside stimuli to entrain and tune the oscillator to adapt to environmental changes. Mutations in circadian genes can lead to mistiming of circadian rhythm, disrupting physiological processes such as sleep, metabolism, and endocrine function. Magel2 has many properties that suggest that it modulates circadian rhythm: it is primarily expressed in the suprachiasmatic nucleus where its transcription oscillates in phase with clock-controlled genes, mice lacking *Magel2 *have disrupted circadian rhythm and metabolic and endocrine deficits, and other members of the Magel2 protein family are directly or circumstantially implicated in circadian rhythm through mouse knockout studies or physical interactions with core circadian proteins. Notably, *Magel2 *is not expressed in peripheral tissues that maintain a circadian cycle, such as the liver, so Magel2 is not required for the cellular maintenance of circadian rhythm *per se*. Moreover, *Magel2 *is not typically identified in screens that depend on gene expression in more than one tissue (e.g. SCN and liver) or that examine cell lines of non-neuronal origin: Magel2 is not expressed in NIH3T3 or HEK293 cells (data not shown). In this study, we explored interactions between Magel2 and the primary circadian feedback loop, involving positive regulation by Clock:Bmal1 heterodimers that initiate transcription of clock-controlled genes, and negative regulation by Per:Cry heterodimers that repress their own transcription by inhibiting Clock:Bmal1-dependent transcription.

We examined several possible mechanisms for the repressive effect of Magel2 on Clock:Bmal1 transcriptional activation, focusing on interactions with circadian components, and effects on sub-cellular localization and stability of core circadian proteins. Consistent with a role as a negative regulator of intrinsic circadian rhythm, Magel2 represses Clock:Bmal1-dependent transcription at a Per2 E-box promoter. In principal, Magel2 may act at the target promoter, alter Clock or Bmal1 independent of promoter binding, or modify the activity of another protein that influences Clock:Bmal1 activity. Furthermore, the repressive function could occur either in the nucleus or in the cytoplasm, as the activity of proteins located in either subcellular compartment can influence activation of clock-controlled genes [36,37,39]. Moreover, the relative abundance of circadian proteins and their subcellular location depends on the circadian time. Between CT 6-10, Clock:Bmal1 is most transcriptionally active in the SCN, with the peak of expression of many clock controlled genes occurring towards the end of this interval. Per2, Atp6a, and Ccr4 transcription peaks around CT10, and Magel2 at around CT14, preceding the expression of Bmal1 from CT16-21 [6,11]. While Clock is constitutively expressed, its endogenous subcellular localization undergoes a circadian oscillation in the SCN [36]. Interestingly, Clock protein is mostly nuclear at CT13 and mostly cytoplasmic by CT19, when Magel2 protein is expected to be most abundant. Our observation that expressing Magel2 in Clock-transfected cells increases the abundance of Clock in the cytoplasm, and that Magel2 and Clock have overlapping immunofluorescence profiles in the cytoplasm of co-transfected cells suggests that Magel2 could be involved in retaining or stabilizing Clock in the cytoplasm around CT19. At this time, cytoplasmic Bmal1 protein levels begin to rise, and Bmal1-mediated nuclear translocation of Clock:Bmal1 complexes occurs. Consistent with this scenario, expression of Magel2 in Clock:Bmal1 doubly-transfected cells did not promote the accumulation of Clock in the cytoplasm, presumably because Bmal1 is more strongly promoting the nucleus-directed movement of Clock. We also observed overlap in the fluorescence signals of Magel2 and Bmal1, and co-immunoprecipitated Magel2 and Bmal1 in co-transfected cells. The transition from Magel2-mediated cytoplasmic retention of Clock to Bmal1-mediated nuclear translocation of Clock may thus involve a physical association between Magel2 and Bmal1. Thus, Magel2 could increase the robustness of the programmed delay in the feedback loop, by modulating Clock:Bmal1 interactions in the cytoplasm prior to the nuclear accumulation of the Clock:Bmal1 heterodimer.

Our co-immunoprecipitation experiments also detected an interaction between Magel2 and Per2, and analysis of immunofluorescence images of Magel2:Per2 co-transfected cells revealed overlap in the immunofluorescence signals in some, but not all cells. The transcription of Per2 occurs from around CT10, but the repressive activity of the Per:Cry heterodimer in the nucleus is delayed by the activities of F-box associated E3 ubiquitin ligases that regulate the transient accumulation of Cry and Per proteins in the cytoplasm, forming a rate-limiting step in the circadian feedback loop [40]. Indeed, mutations in the genes encoding subunits of these phosphorylation and degradation complexes cause circadian defects in mice [14,41]. MAGE proteins are adaptors for E3 ubiquitin ligase complexes, but the activity of Magel2 in these complexes remains enigmatic. The interaction of Magel2 with Per2 may add robustness to the negative limb of the feedback loop through interactions that promote transient Per2 retention or stability in the cytoplasm. However, we did not see a Magel2-dependent accumulation of Per2 in the cytoplasm, and expression of Magel2 does not alter the stability of Per2, arguing against this role for Magel2 in the cytoplasm. Future studies that examine interactions of Magel2 with RING proteins implicated in the degradation of circadian proteins may shed further light on this process. Alternatively, Magel2 could interact with Per2 while it inhibits Clock:Bmal1 in the nucleus, as we did observe overlap of the Magel2 and Per2 immunofluorescence signals in the nucleus in co-transfected cells. Other possible relationships between Magel2 and circadian proteins have not yet been explored. The MAGE protein necdin regulates the acetylation status and activity of p53 through interactions with SIRT1, and the acetylation and activity of Per2 is also modulated by SIRT1, and Magel2 could take part in this regulation. Another MAGE protein, Maged1, acts in a second auto-regulatory loop involving the Clock:Bmal1 activated transcription of retinoic acid-related orphan nuclear receptors *Rev-erbα *and *Rorα*., by cooperating with *Rorα *to modulate the expression of circadian genes, including *Bmal1 *and *Rev-erbα *[34] through response elements in their promoters, but a parallel role for Magel2 has not been explored.

Although a small handful of extensively studied proteins drive core circadian rhythm feedback loops, hundreds of additional proteins control the robustness of this rhythm to compensate for external influences [42]. It has proven challenging to integrate these novel components into the molecular circuitry that drives the rhythm, particularly for proteins whose function is poorly understood. As Magel2 is not expressed in the mouse liver, mouse embryonic fibroblasts, or in many cultured cell lines typically used for studies of circadian proteins, our study was limited to the examination of heterologously expressed epitope-tagged proteins. Further molecular characterization of Magel2 in circadian pathways, and continued analysis of circadian defects in the *Magel2*-null mouse strain will elucidate the contribution of this enigmatic protein to mammalian circadian rhythm. In particular, it will be important to determine whether Magel2 co-localizes with circadian proteins in SCN neurons, and whether the loss of the endogenous Magel2 protein affects the activity of the Clock:Per2 heterodimer in SCN neurons, to elucidate the role of Magel2 in the intrinsic circadian rhythm versus its potential developmental role in the hypothalamus. These studies will also add to our understanding of the role of normal circadian rhythm in human health, and the etiology of circadian rhythm dysfunction in human genetic disorders such as Prader-Willi syndrome.

## List of abbreviations

bHLH: basic helix-loop-helix; CS: calf serum; CT: circadian time; Cry: Cryptochrome; MAGE: melanoma antigen; PAS: Per/Arnt/Sim; Per: Period; PWS: Prader-Willi syndrome; SCN: suprachiasmatic nucleus.

## Competing interests

The authors declare that they have no competing interests.

## Authors' contributions

SVW and JD performed and analyzed all experiments and helped draft the manuscript. RW conceived of and supervised the study and completed writing the manuscript. All authors read and approved the final manuscript.
